# Changes in melon plant phytochemistry impair *Aphis gossypii* growth and weight under elevated CO_2_

**DOI:** 10.1038/s41598-021-81167-x

**Published:** 2021-01-26

**Authors:** Ana Moreno-Delafuente, Ignacio Morales, Elisa Garzo, Alberto Fereres, Elisa Viñuela, Pilar Medina

**Affiliations:** 1 Unidad de Protección de Cultivos, Departamento de Producción Agraria, Escuela Técnica Superior de Ingeniería Agronómica, Alimentaria y de Biosistemas, Universidad Politécnica de Madrid, 28040 Madrid, Spain; 2 Insectos Vectores de Patógenos de Plantas, Departamento de Protección Vegetal, Instituto de Ciencias Agrarias, Consejo Superior de Investigaciones Científicas, 28006 Madrid, Spain; 3 Associate Unit IVAS (CSIC-UPM), Control of Insect Vectors of Viruses in Horticultural Sustainable Systems, Madrid, Spain

**Keywords:** Plant sciences, Zoology, Environmental sciences

## Abstract

Elevated CO_2_ (eCO_2_) modifies plant primary and secondary metabolism that subsequently impacts herbivore insect performance due to changes in its nutritional requirements. This laboratory study evaluated interactions between *Aphis gossypii* Glover (Hemiptera: Aphididae) and melon (*Cucumis melo* L., Cucurbitaceae), previously acclimated two or six weeks to different CO_2_ levels, eCO_2_ (700 ppm) or ambient CO_2_ (400 ppm). Under eCO_2_, melon plants decreased nitrogen foliar concentration and increased carbon to nitrogen ratio, independently of acclimation period, significantly reducing the content of some amino acids (alanine, asparagine, glycine, isoleucine, lysine, serine, threonine, and valine) and increasing the carbohydrate (sucrose) content in melon leaves. The dilution in some essential amino acids for aphid nutrition could have aggravated the reduction in *A. gossypii* population growth reared on melon previously acclimated two weeks to eCO_2_, as well as the loss of aphid body mass from two successive generations of *A. gossypii* reared under eCO_2_ on plants previously acclimated two or six weeks to eCO_2_. The response to eCO_2_ of phloem feeders, such as aphids, is actually variable, but this study highlights a negative response of *A. gossypii* to this climate change driver. Potential implications on control of this pest in a global change scenario are discussed.

## Introduction

Anthropogenic activities, such as changes in land use and increased fossil fuel burning and deforestation, are the main responsible for carbon dioxide (CO_2_) emissions. The atmosphere concentration of CO_2_ would be projected to increase from the current level above 411 ppm in 2020^1^ to about 670 ppm by the end of the century, contributing to a global warming over 2.2 °C, according to the RCP6.0 future climate scenario^2^. Apart from the rise in temperature, the increase in CO_2_ will result in changes in rainfall and weather patterns, which directly affects agricultural systems^3,4^. Consequently, growth and physiological functions of plants are seriously affected by elevated CO_2_ (eCO_2_)^5^. Some positive effects of eCO_2_ are related with the stomatal closure in plant tissues, which reduces transpiration, improves water conservation and leads to higher photosynthesis rates, increasing biomass, yield and plant growth^6–10^. Elevated CO_2_ modifies plant metabolism, both the primary (nitrogen, proteins, water, soluble sugars, starch and structural compounds) and the secondary (terpenes, tannins, phenolics and total non-structural carbohydrates)^7^. Due to CO_2_ enrichment, plant tissues and sap normally decrease nitrogen (N) content and increase or maintain carbon (C) content, finally rising C:N ratio in plants^11–14^. In addition to the decrease in the total amount of N (N quantity), eCO_2_ also modifies the composition of nitrogenous compounds (N quality)^15^, such as amino acids and proteins. Furthermore, eCO_2_ induces the accumulation of non-structural carbohydrates, for example starch and soluble sugars, in plants^7^.

Because N is a limiting nutrient for herbivores^15^, eCO_2_ indirectly impacts herbivorous insect pest performance^7,16,17^. Free amino acids are the principal nitrogenous compounds in phloem sap^18^; among them, there are nine essential amino acids which animals cannot synthesize de novo: histidine, isoleucine, leucine, lysine, methionine, phenylalanine, threonine, tryptophan, and valine^19^. Except leucine, the other eight amino acids are essential for aphids^20^, although the requirements of some amino acids could change among aphid species, even among aphid clones^19,20^. Aphids complete their N requirements not only from phloem sap ingestion but also from the symbiotic bacteria of the genus *Buchnera*, which also provides these essential amino acids^18^. Elevated CO_2_ usually decreases amino acids content in plants modifying differently aphids’ development, fecundity, longevity, honeydew production, population dynamics, etc^11,12,19–25^. However, a significant increase in free amino acids content has also been observed in other plants under eCO_2_^20,23^_._

Non-structural carbohydrates can act as phagostimulants^7^, i.e. compounds that tasted by aphids can stimulate and sustain its feeding^26^. Sucrose is a major transport sugar, the most abundant carbohydrate in phloem sap and the most effective phagostimulant for herbivorous insects. Numerous species from Cucurbitaceae family also transport raffinose, stachyose and higher order oligosaccharides. Polyols (sugar alcohols) are also abundant in phloem^18^. However, sugars are not limiting nutrient source for aphid feeding^15,18^. The effect of eCO_2_ on plant carbohydrate content is species-specific thus, most of the plants show an increase in carbohydrates content^14,20,27^ meanwhile in others, soluble sugars are not affected by eCO_2_^24^.

In general, the increase in plant biomass and the accumulation of C-based compounds due to eCO_2_ could dilute the concentration of foliar proteins; finally counteracting the positive effect that the boost in phagostimulatory activity due to carbohydrates increment produces in herbivorous insects^7^.

Aphids are very sensitive to changes in quality and quantity of their nutritional requirements^24^. Therefore, aphid responses to eCO_2_–mediated effects on host plant quality and quantity nutrient compounds are particularly variable, and could be either positive^22,28,29^, negative^12,13,24,30,31^ or not significantly modified^17,25^, comparing to aphid performance under current CO_2_ concentration.

Most of the studies investigating the effects of eCO_2_ on agricultural crops have been focused on grains, predominantly cereals but also some legumes^32^. However, few studies have analyzed the effect of climate change on plant–herbivore interactions in horticultural crops^13,33^. This is the first time a research focused on the impact of increasing atmospheric CO_2_ on melon plants (*Cucumis melo* L., Cucurbitaceae) under a climate change scenario. The cotton aphid, *Aphis gossypii* Glover (Hemiptera: Aphididae) is one of the principal pest species colonizing almost one hundred of plant species, actually one of the most important aphid pests on cucurbits. *Aphis gossypii* is originated from warmer regions, but can also survive northern winters in greenhouses^34^. Milder winters under climatic change could increase winter survival of insect pests and rates of herbivory^3^, therefore intensifying the damage of cotton aphid. Elevated CO_2_ has been found to affect *A. gossypii* feeding on cotton (*Gossypium hirsutum* L.), ingesting more phloem sap due to a higher plant C:N ratio and lower levels of amino acids, although no change in the mean relative growth rate was found when compared eCO_2_ to ambient CO_2_ (aCO_2_)^25^.

The objectives of our study were to analyze: (1) if eCO_2_ changes melon plant biomass and biochemistry, specifically amino acids and soluble carbohydrates content; (2) in consequence, if eCO_2_ mediated changes on plants could affect aphid performance, and; (3) whether a longer acclimation period to eCO_2_ could impact more severely both plants and aphids. For that purpose, we analyzed the effect of eCO_2_ on *A. gossypii* body mass and colony growth rate, reared on melon plants previous acclimated during two or six weeks to different CO_2_ regimes, eCO_2_ (700 ppm) or aCO_2_ (400 ppm).

## Materials and methods

### Melon plants and aphids

Biological material production and experiment setup were conducted in the Institute of Agricultural Sciences of the Spanish National Research Council (ICA-CSIC, Madrid, Spain). Melon cv. Sancho (Syngenta Seeds B.V., Enkhuizen, The Netherlands) plants were used in the experiments. After germination in darkness above wet filter paper in a Petri dish, seedlings were transplanted at one week old with a mixture of equal parts of soil substrate (GoV4, Jiffy International, A.S. Norway) and vermiculite (No. 3, Asfaltex S.A., Barcelona, Spain) to 11 × 11 × 12 cm pots. Plants were placed since seedling in the plant growth chamber at 24:20 °C temperature, 60:100% RH and 16:8 h (L:D) photoperiod until CO_2_ acclimation. Plants were watered on alternate days (680 mL/plant-week). A NPK 20-20-20 fertilizer (Miller Chemical & Fertilizer Corp., Pennsylvania, USA) was added to the irrigation water (1 g/L).

The clonal *A. gossypii* colony at the laboratory was initiated from a single virginiparous apterae collected from melon in El Ejido, Spain, in 1998. Aphid colonies were reared on melon plants for several generations inside rearing cages in environmental growth chamber at optimal development conditions of 23:18 °C temperature, 60–80% RH and 14:10 h (L:D) photoperiod. Aphids were synchronized prior the bioassays to guarantee age homogeneity (10–11 days old) at the time of the experiment.

### Plant acclimation to CO_2_

Two walk-in climate chambers were used for plant acclimation to CO_2_ with identical conditions of 24:20 °C temperature, 60–70% RH, 14:10 h (L:D) photoperiod, and 310 ± 3 µmol m^−2^ s^−1^ light intensity at canopy level (GreenPower LED production dr/b/fr 150, Philips, Eindhoven, The Netherlands); but with different CO_2_ atmospheric concentrations, one chamber with eCO_2_—700 ppm (703.28 ± 1.81 ppm) and the other with aCO_2_—400 ppm (409.89 ± 1.40 ppm). Temperature and humidity data were recorded every hour with a data logger (Tinytag Ultra 2, Gemini Data Loggers, UK) in each chamber. CO_2_ concentration was monitored in aCO_2_ chamber with a datalogger device (Rotronic AG CP11, Bassersdorf, Swirtzeland), while eCO_2_ chamber incorporated a system that automatically regulated and recorded the chamber gas concentration.

One-week-old melon plants were divided into four sets and two of them were placed in eCO_2_ or aCO_2_ chamber respectively for six weeks of acclimation period, whereas the remaining two sets were maintained in the general plant growth chamber (see conditions above). Two weeks before the beginning of plant measurements, these sets were transferred to eCO_2_ and aCO_2_ chambers respectively for two weeks of acclimation period^35^. All plants were 7-weeks-old when experiments started and insect experimental units were maintained in their respective CO_2_ treatment chambers during the bioassays.

### Plant measurements

#### Total carbon and nitrogen concentration

When the previous acclimation to two or six weeks to aCO_2_ or eCO_2_ respectively was concluded, five plants per treatment were randomly collected for destructive sampling to assess the effects of plant exposure to CO_2_ on total C and N plant concentration. Melon stems and leaves were analyzed separately as plant chemical composition can differ within plants, and these specific niches could subsequently affect aphid performance in a different manner^36^. Stems and leaves separately were cut (pieces of 1–2 cm) and dried in a drying-oven (Selecta, Barcelona, Spain) for 48 h at 60 °C. They were then milled into powder with an analytical grinder (YellowLine A10, IKA-WERKE, Germany). Total C and N concentration was determined using an Organic Elemental Analyzer–NC Soil Analyzer (Flash 2000, Thermo scientific, Waltham, USA)^37^ at the Analysis of Soils, Plants and Waters Service in ICA-CSIC. C:N ratio was calculated by dividing the concentration of C by the concentration of N for each sample. This experiment was repeated twice, obtaining finally ten replicates of leaf and stem samples respectively.

#### Plant biomass

To assess the effect on plant weight to the exposure of CO_2_, ten plants per treatment were randomly collected for destructive sampling when the previous acclimation period to two or 6 weeks to aCO_2_ or eCO_2_ was concluded. Plants were separated in stems and leaves, then, samples were maintained at − 20 °C and the day before the freeze-drying, they were deep-frozen at − 80 °C. Once freeze-dried (Epsilon 2–4 LSCplus freeze dryer, Christ, Osterode am Harz, Germany), samplings were weighed on an analytical balance (model AB204, Mettler Toledo, Greifensee, Switzerland) to calculate their dry weight and kept in a desiccator to analyze amino acids and carbohydrates pigments at a later time.

#### Amino acids and carbohydrates content

Free amino acids and carbohydrates were obtained adapting the extraction method and the Gas Chromatography Mass Spectrometry analysis of plant samples (n = 6), leaves and stems separately, from the protocol described on supplementary information in Corrales et al*.*^38^, at the Metabolomic Service in Centro de Biotecnología y Genómica de Plantas (CBGP, UPM-INIA, Madrid, Spain). Amino acids and carbohydrates were measured from tissue extraction, instead of phloem sap collection that could be a priori better related with sap-feeding insects, after investigating that tissue extraction has been shown to be a reliable indicator on the relative composition of amino acids and some carbohydrates (e.g. sucrose) in other crops, such as lucerne (*Medicago sativa* L.)^21,23^, barley (*Hordeum vulgare* L.)^39^, and spinach (*Spinacia oleracea* L.)^40^.

#### Aphid growth and performance

##### Effects of CO_2_ on *Aphis gossypii* adult weight

In order to calculate aphid body mass, we weighed plots of 50 synchronized first (F1) and second (F2) generation adults (7-days-old), exposed to the different CO_2_ concentration on melon plants previously acclimated to aCO_2_ or eCO_2_ for 2 or 6 weeks. To get F1 *A. gossypii* adults, the day that the previous plant acclimation period to two or six weeks to aCO_2_ or eCO_2_ concluded*,* 100 adults of *A. gossypii* from the synchronized rearing, were placed distributed in 18 clip-cages in a plant of each treatment. Twenty-four hours later, adults were removed and onset nymphs were left to develop themselves exposed to the CO_2_ conditions determined for each treatment. Seven days later, when nymphs had already reached adulthood (F1 adults), we proceeded to weigh them. To get F2 adults, 100 adult aphids from the first generation of each treatment were placed in another acclimated plant of the same treatment. Twenty-four hours later, adults were removed and onset nymphs (start of the *A. gossypii* second generation) were left to develop themselves exposed to the different conditions of CO_2_. Seven days later, when nymphs had already reached adulthood, we proceeded to weigh F2 adults. For each aphid generation, groups of 50 adults (n = 12) were made to calculate aphid average weight. Adults were anesthetized with CO_2_ and then weighed (fresh weight) on an analytical balance (Mettler AE166 DeltaRange, Greifensee, Switzerland). Then, samples were put in an oven-drier at 60 °C for 24 h and weighed again (dry weight).

##### Effects of CO_2_ on *Aphis gossypii* colony performance

The day of the beginning of bioassays, when melon plants had been previously acclimated two weeks to aCO_2_ or eCO_2_, two synchronized adults (12-days-old) were placed in each plant (ten plants per treatment), in order to acclimate aphids to the respective CO_2_ concentration. Twenty-four hours later, aphids were removed except six nymphs per plant. Each plant was covered with a fine mesh, to facilitate aphid dispersal in the plant but avoiding contamination between plants. After 7 days, when nymphs had already reached adulthood, only two were left per plant. The offspring of these previously acclimated adult females were counted at 14 and 21 days. For the last count, samples were frozen to facilitate a later counting due to the massive number of aphids. The aphid colony growth rate on each plant was calculated as the difference between number of aphids on a given day and the number of aphids on the previous count day^30^. In our case, analysis was performed with the increase in population number from day 7 to 14, and from day 14 to 21, so this value was calculated between weeks.

##### Statistical analysis

To determine the effects of CO_2_ concentration, acclimation period to CO_2_ and their interaction, all plant data (C and N concentration, biomass, amino acids and carbohydrates content) and aphid adult weight data were subjected to the two-way analysis of variance (ANOVA) using the General Linear Model module in IBM SPSS Statistics 22.0.0.0 software (package for Windows, 64-bit edition, Chicago, USA). Whenever interaction between factors was statistically significant (P < 0.05), a post hoc LSD test was performed for pairwise comparisons. To achieve normality and homoscedasticity of some parameters, data was transformed by sqrt(x + 0.5) or log(x + 1). *Aphis gossypii* colony performance were analyzed by Student *t*-test (P ≤ 0.05) with the same statistical software.

## Results

### Total carbon and nitrogen concentration

**Figure 1:**
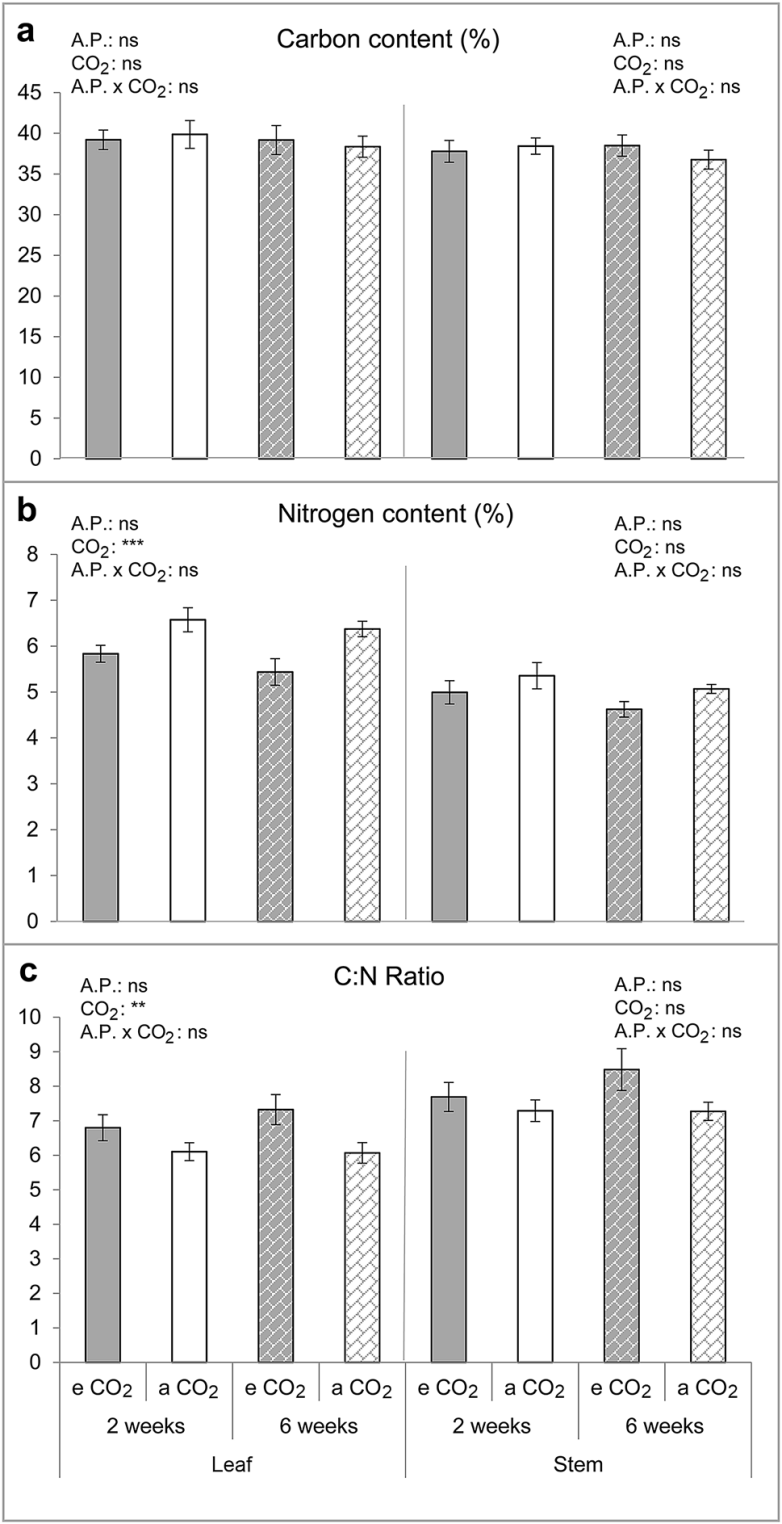
Melon carbon and nitrogen concentration profile. (**a**) Carbon (C) concentration (%), (**b**) Nitrogen (N) concentration (%) and, (**c**) C:N ratio from leaves and stems of melon plants measured after a previous acclimation period (A.P.) to two or six weeks to elevated CO_2_ (eCO_2_) (700 ppm) or ambient CO_2_ (aCO_2_) (400 ppm). Mean values ± SE are shown (n = 9). **(P ≤ 0.01) and ***(P ≤ 0.001) when statistically significant differences were found, ns (no statistically significant differences) (Two-way ANOVA and LSD tests). Melon leaf C:N ratio data were transformed by log (x + 1).

Nitrogen concentration in melon leaves was significantly affected by CO_2_ concentration, being significantly lower under eCO_2_ than under aCO_2_ (F_1,32_ = 13.065; P = 0.001), whereas C concentration in melon leaves was not affected by the CO_2_ concentration level (F_1,32_ = 0.003, P = 0.959). Statistically significant differences were also found in C:N ratio in melon leaves due to CO_2_ concentration. A significant increase in foliar C:N ratio due to the dilution in N concentration occurred under eCO_2_ compared to aCO_2_ (F_1,32_ = 7.873; P = 0.008) (Fig. 1, Supplementary Table S1). In contrast, acclimation period did not affect C nor N concentration in melon leaves.

Neither the C and N concentration nor C:N ratio in melon stems were significantly affected by CO_2_ concentration or acclimation period (Fig. 1, Supplementary Table S1).

### Plant biomass

**Figure 2:**
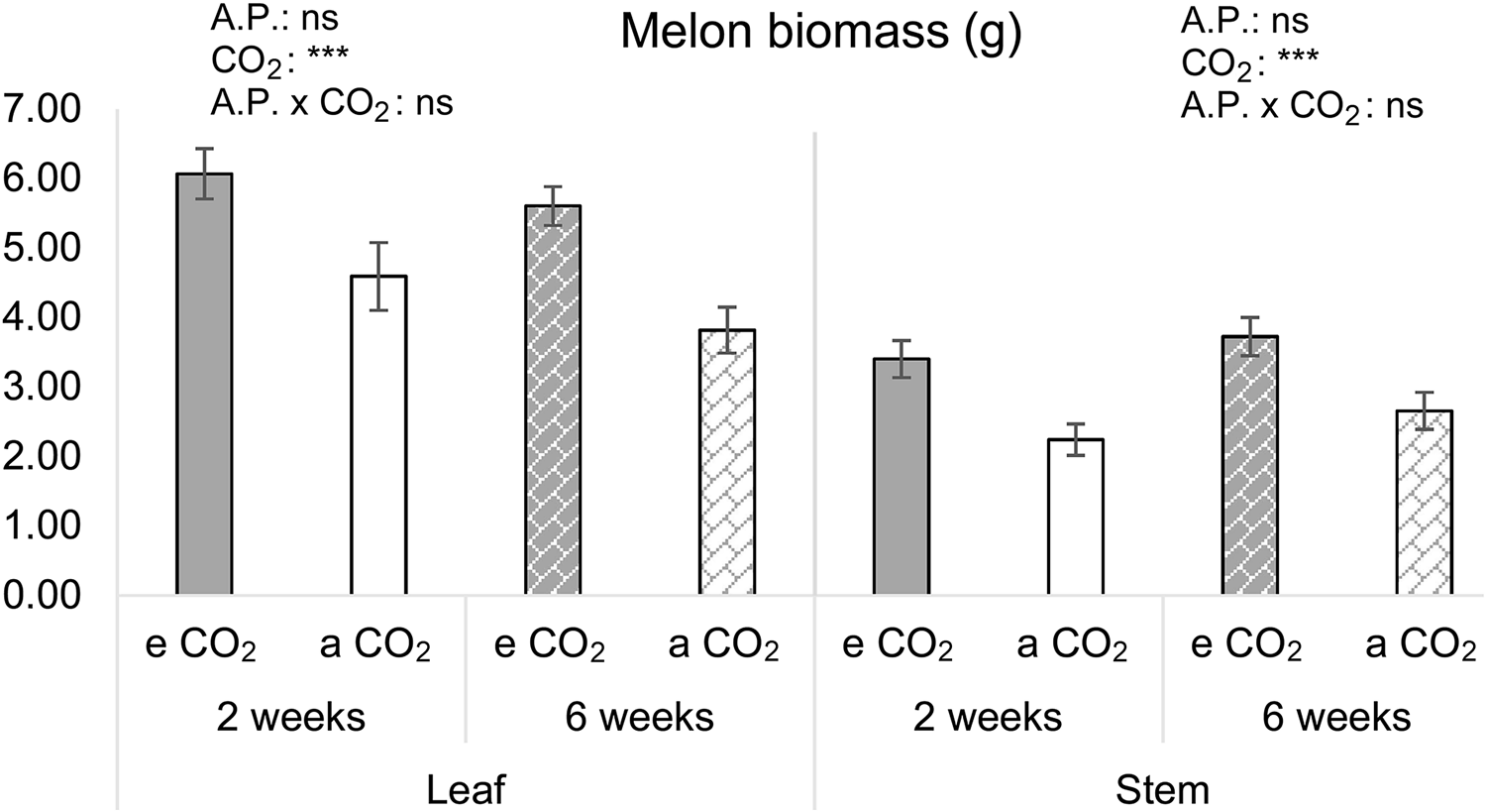
Melon biomass. Dry weight of leaves and stems of melon plants measured after a previous acclimation period (A.P.) to two or six weeks to elevated CO_2_ (eCO_2_) (700 ppm) or ambient CO_2_ (aCO_2_) (400 ppm). Mean values ± SE are shown (n = 10). ***(P ≤ 0.001) when statistically significant differences were found, ns (no significant differences) (Two-way ANOVA and LSD tests).

Leaves and stems biomass significantly increased under eCO_2_ compared to under aCO_2_ (Leaves dry weight: F_1,35_ = 19.016, P < 0.001; Stems dry weight: F_1,35_ = 18.354, P < 0.001), but none statistically significant effect was observed due to the acclimation period (Fig. 2; Supplementary Table S1).

### Amino acids content

**Figure 3:**
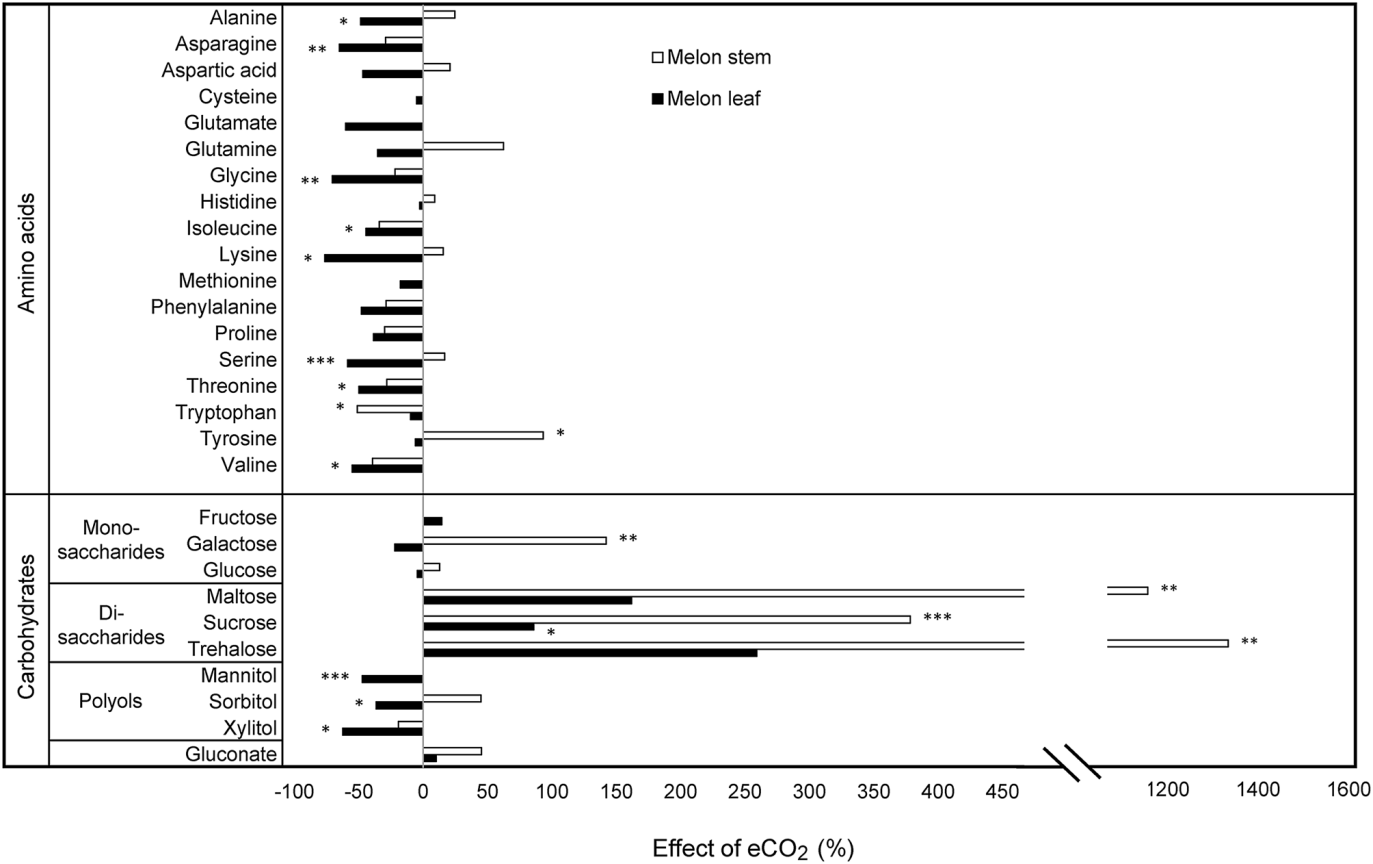
Relative effect of elevated CO_2_ on the content of amino acids and carbohydrates on leaves and stems of melon plants. Bars represent the percentage change value between elevated CO_2_ (eCO_2_) (700 ppm) and ambient CO_2_ (aCO_2_) (400 ppm) by the formula: percentage change value (%) = ((eCO_2_—aCO_2_)/aCO_2_) × 100. Results of the effect of CO_2_ concentration (melon leaf and stem separately) by Two-way ANOVA and LSD tests are denoted by asterisks: *(P ≤ 0.05), **(P ≤ 0.01) and ***(P ≤ 0.001) when statistically significant differences were found.

**Figure 4:**
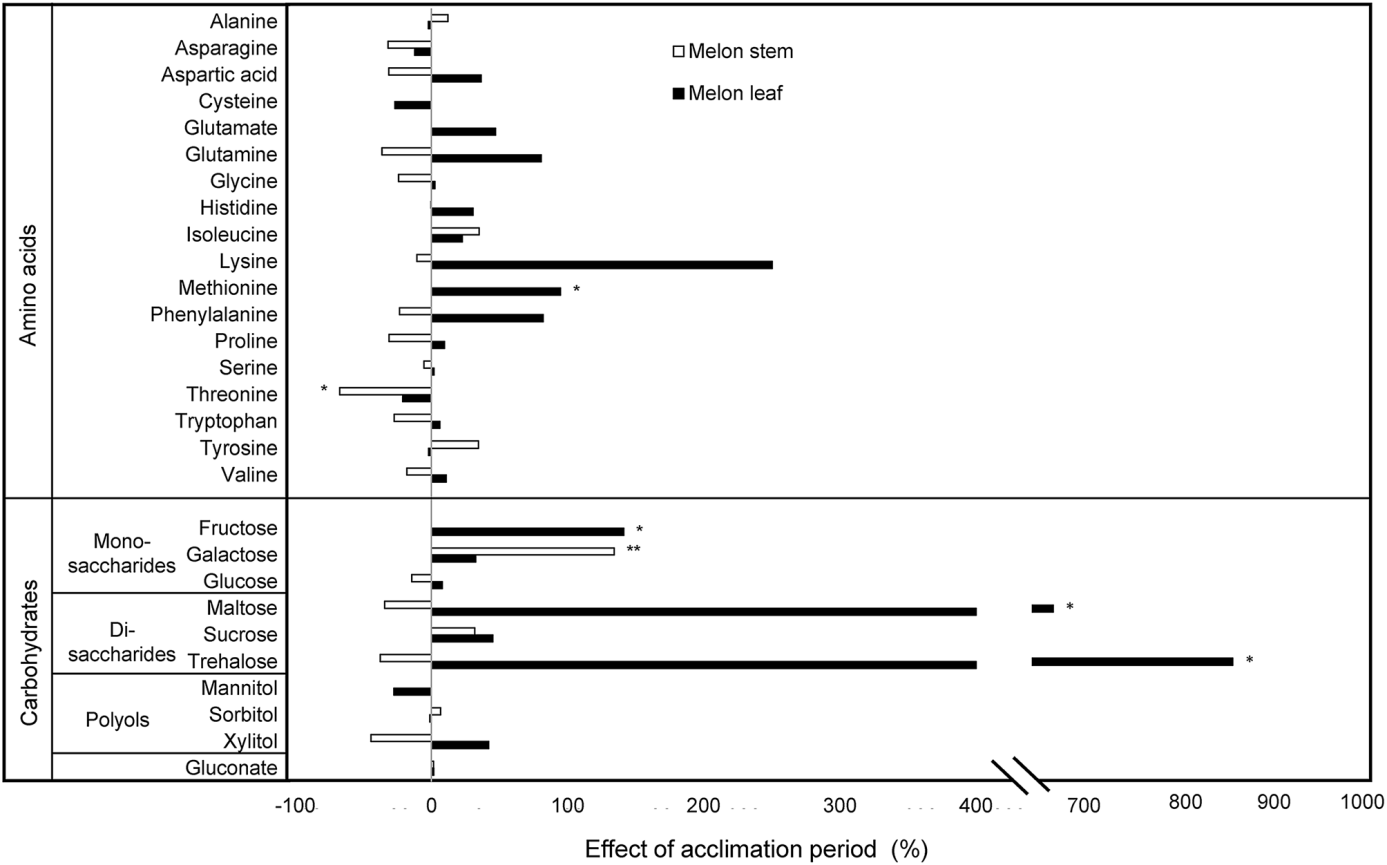
Relative effect of six weeks of acclimation period on the content of amino acids and carbohydrates on leaves and stems of melon plants. Bars represent the percentage change value between 6 weeks (6w) of acclimation period (A.P.) and 2 weeks (2w) of A.P. by the formula: percentage change value (%) = ((6w – 2w)/ 2w) × 100. Results of the effect of A.P. (melon leaf and stem separately) by Two-way ANOVA and LSD tests are denoted by asterisks: *(P ≤ 0.05) and **(P ≤ 0.01) when statistically significant differences were found.

In total, 18 individual amino acids were detected in melon leaves, whereas only 15 were determined in melon stems. Elevated CO_2_ compared to aCO_2_ significantly decreased the concentration of Alanine (49%), Asparagine (65%), Glycine (71%), Isoleucine (44%), Lysine (76%), Serine (59%), Threonine (50%), and Valine (55%) in melon leaves (Fig. 3, Supplementary Table S2). Methionine significantly increased its concentration by 95% under 6 weeks of acclimation period compared to 2 weeks (Fig. 4, Supplementary Table S2).

In melon stems, effects of both factors CO_2_ and acclimation period were less pronounced than in melon leaves. Elevated CO_2_ significantly increased Tyrosine content (93%), whereas Tryptophan content was reduced under eCO_2_ (51%) compared to aCO_2_ (Fig. 3, Supplementary Table 2). After 6 weeks of acclimation period a significant reduction of Threonine content (67%) was scored compared to 2 weeks of acclimation period (Fig. 4, Supplementary Table S2). The interaction between CO_2_ concentration and acclimation period was significantly different on Asparagine content in melon stems (Supplementary Table S2).

### Carbohydrates content

The content of sugars on melon leaves and stems was significantly affected by CO_2_ concentration or by acclimation period to CO_2_. There was no interaction between the two factors (Supplementary Table S3). Elevated CO_2_ compared to aCO_2_ significantly increased the concentration of sucrose (86%) and significantly decreased the concentration of mannitol (47%), sorbitol (37%) and xylitol (63%) in melon leaves (Fig. 3, Supplementary Table S3). Fructose, maltose and trehalose were significantly affected by acclimation period on melon leaves, increasing their concentration by 141%, 650% and 854% respectively after 6 weeks of acclimation period compared to 2 weeks (Fig. 4, Supplementary Table S3).

Galactose, maltose, sucrose and trehalose significantly increased their content on melon stems under eCO_2_ compared to aCO_2_ by 142%, 1157%, 378% and 1334%, respectively (Fig. 3, Supplementary Table S3). Galactose was also affected by acclimation period to CO_2_, increasing its content by 134% after 6 weeks of acclimation period compared to 2 weeks (Fig. 4, Supplementary Table S3).

### Effects of CO_2_ on *Aphis gossypii* adult weight

**Figure 5:**
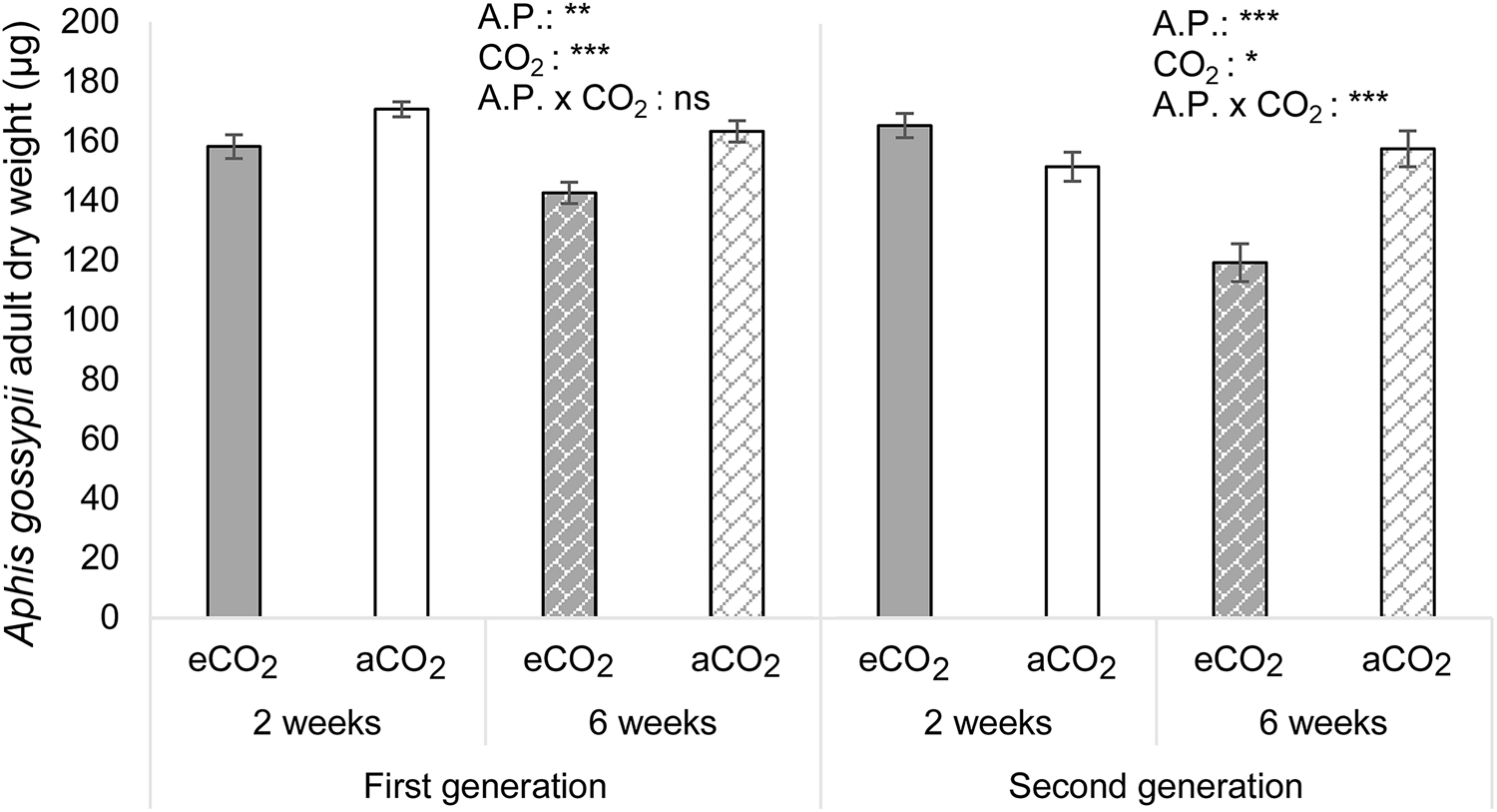
*Aphis gossypii* body mass. Dry weight (µg) of adult *Aphis gossypii* (mean ± SE) developed under ambient (aCO_2_) (400 ppm) or elevated (eCO_2_) (700 ppm) CO_2_, on melon plants previous acclimated for 2 or 6 weeks to the respective CO_2_ concentration. Values are the average of sets of 50 synchronized adults (n = 12). First and second generation were examined separately*.* *(P ≤ 0.05), **(P ≤ 0.01) and ***(P ≤ 0.001) when statistically significant differences were found, ns (no significant differences) (Two-way ANOVA and LSD tests). A.P. = Acclimation Period to the respective CO_2_ concentration.

Dry body mass of F1 *A. gossypii* adults was significantly affected by the CO_2_ concentration level and by the acclimation period of 2 and 6 weeks. F1 aphid body mass significantly decreased under eCO_2_ compared to aCO_2_ (F_1,43_ = 23.044, P ≤ 0.001). Furthermore, F1 aphid body mass was significantly lower when aphids fed on plants previous exposed to a longer acclimation period of 6 weeks compared to the shorter acclimation period of 2 weeks (F_1,43_ = 10.940, P = 0.002) (Fig. 5).

Dry body mass of F2 *A. gossypii* adults was significantly affected by the CO_2_ concentration level depending on the acclimation period of 2 and 6 weeks (F2: acclimation period × CO_2_: F_1,41_ = 22.992, P ≤ 0.001). Due to the significant interaction, data was analysed by LSD pairwise comparison. There was a significant decrease in the body mass when aphids fed on melon plants previously acclimated for 6 weeks to eCO_2_ (119.27 ± 6.29 µg) compared to 2 weeks under eCO_2_ (165.27 ± 4.14 µg). Furthermore, there was a significant loss of weight on aphids grown on melon plants previous acclimated 6 weeks under eCO_2_ (119.27 ± 6.29 µg) than under aCO_2_ (157.50 ± 6.01 µg) (Fig. 5).

Consequently, the dry weight of the aphid grown on plants previously acclimated during 6 weeks at eCO_2_ decreased for both *A. gossypii* generations, whereas this effect was more pronounced in the second generation.

### Effects of CO_2_ on *Aphis gossypii* colony performance

**Figure 6:**
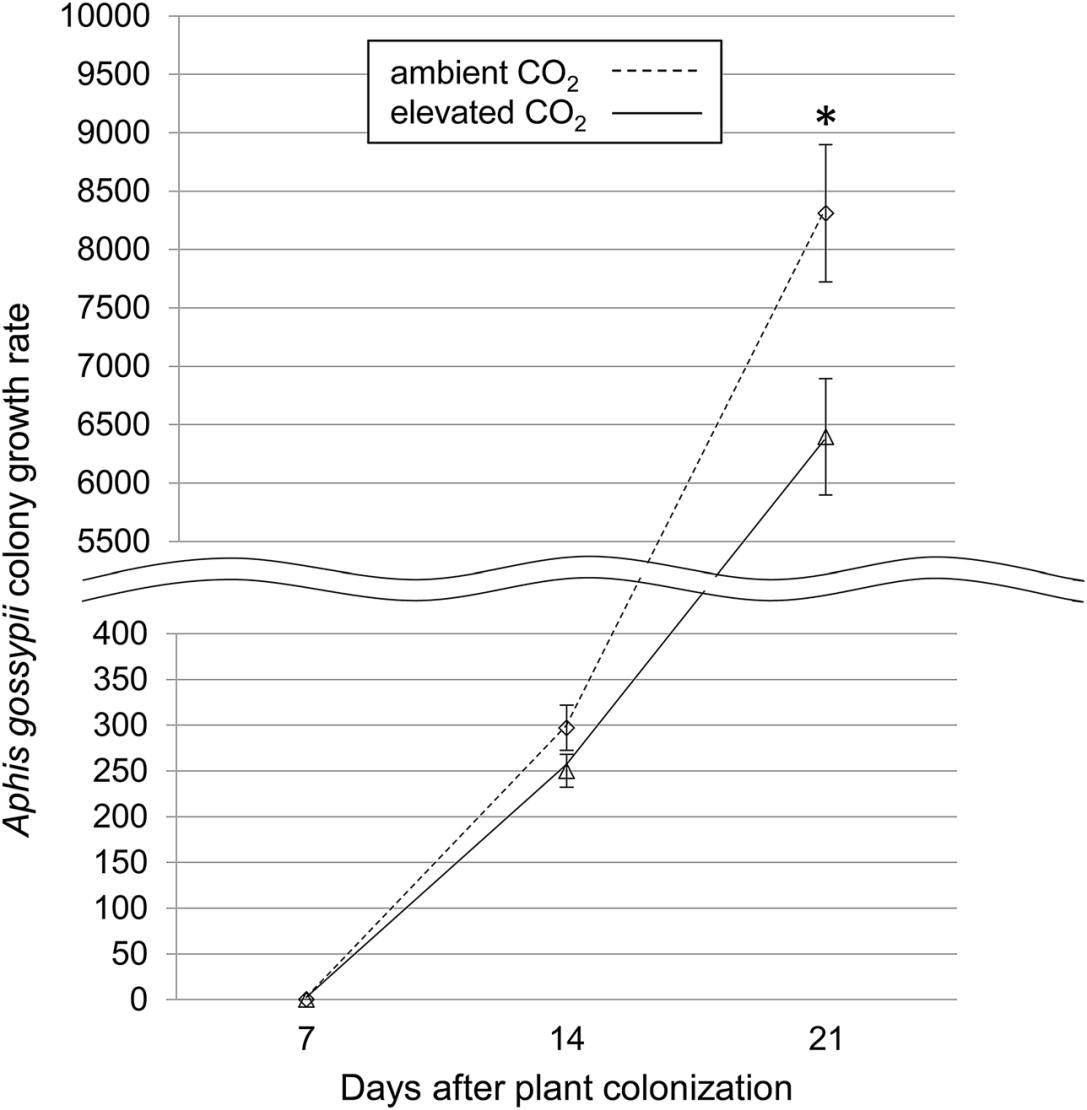
Colony growth rate of *Aphis gossypii*. The mean (± SE) of the colony growth rate was measured as the sum of nymphs and adults of *Aphis gossypii* developed under ambient (400 ppm) or elevated CO_2_ (700 ppm). Each colony was generated from two adult females reared on melon plants acclimated for two weeks to the respective CO_2_ concentration. Growth rates were calculated weekly (day 14 as the difference in the number of aphids on day 14 compared to day 7; and day 21, comparing the number of aphids on day 21 to day 14). Ten (n) *A. gossypii* colonies per CO_2_ concentration. P-values based on Student *t*-test (*P ≤ 0.05).

*Aphis gossypii* population performance differed when colony was reared under aCO_2_ or eCO_2_ conditions on plants previously acclimated for 2 weeks to the respective CO_2_ concentration. No statistical differences were observed in day 14 (Colony growth rate: t = − 1.550, df = 18, P = 0.139). However, the number of aphids decreased by 23% in the *A. gossypii* colony under eCO_2_ in day 21, with fewer nymphs number, and subsequently less colony growth rate, compared to the colony developed under aCO_2_ concentration (Nymphs growth rate: t = − 2.675, df = 18, P = 0.015; Colony growth rate: t = − 2.486, df = 18, P = 0.023) (Fig. 6, Supplementary Table S4).

## Discussion

This research emphasizes how the increase in atmospheric CO_2_, main driver of climate change, generates changes in plant nutritional quality and subsequently, influences pest insect performance. We mainly focused on how different plant acclimation to eCO_2_ modified the content of carbohydrates and amino acids of melon plants and affected *A. gossypii* body mass and population growth. Under eCO_2_, melon plants decreased N foliar concentration and increased C:N ratio, independently of acclimation period. Elevated CO_2_ leaded to changes in primary metabolites, significantly reducing the content of some amino acids and increasing some carbohydrates. Few carbohydrates were influenced by acclimation period, increasing their content under longer exposure to experimental climate conditions. Due to the importance of amino acids for aphid nutrition, the dilution of the foliar content of some essential amino acids could have aggravated the reduction in the population growth of *A. gossypii* reared on melon plants previous acclimated two weeks to eCO_2_, and the loss of aphid body mass from two successive generations of *A. gossypii* reared under eCO_2_ on plants previous acclimated 2 or 6 weeks. Furthermore, the drop in aphid body mass was more pronounced when reared longer period under eCO_2_ (6 weeks of acclimation compared to 2 weeks), and more marked in the second generation of *A. gossypii* compared to the first generation.

Atmospheric CO_2_ enrichment usually promotes an increase in plant biomass^6,8,10,13,23,29,32,41^, as occurred in melon plants under eCO_2_ in our experiment. Furthermore, the significant decrease in foliar N concentration and the subsequently increase in C:N ratio in melon plants under eCO_2_ could well explain the drop in *A. gossypii* adult weight and in the colony growth rate, being consistent with other studies that also observed a negative effect of eCO_2_ on aphid performance^13,29,42,43^. However, as explained by Wilkinson & Douglas^19^, the total N content of plant tissue (i.e. the N quantity) normally used to relate the plants nutritional value for herbivores and commonly used to explain plant–herbivore interactions under climate change, may not deeply describe the real dietary requirements needed by the insect, that could be only explained investigating the individual amino acids content (i.e. the N quality).

To go one step forward, according to Wilkinson & Douglas^19^, we used foliar amino acids and carbohydrates in order to relate biochemical compounds with nutritional quality of melon plants for aphids under eCO_2_. In our study, five essential amino acids: isoleucine, lysine, threonine and valine (in leaves) and tryptophan (in stems), as well as other amino acids, such as alanine, asparagine, glycine, and serine (in leaves), reduced their content under eCO_2_, consequently affecting the N quality and therefore, the nutritional value of melon plants for *A. gossypii* reared under these climatic conditions. In consonance with the research performed by Sun et al*.*^25^, alanine, glycine, lysine, threonine and tryptophan content also decreased in cotton phloem sap under eCO_2_, forcing *A. gossypii* to ingest more phloem sap to satisfy its nutritional requirements. Alanine was also significantly reduced, although histidine and tryptophan increased their concentrations, in wheat (*Triticum aestivum* L.) under eCO_2_^27^. Ryan et al*.*^12^ observed that arginine, aspartate (aspartic acid), glutamine and valine in the pasture grass *Schedonorus arundinaceus* Schreb were correlated with *Rhopalosiphum padi* L. abundance, but only valine appeared to decrease due to eCO_2_, explaining to some extent the decrease in aphid performance. Accordingly, *Myzus persicae* Sulzer was negatively affected by the decrease in individual amino acid concentrations in oilseed rape (*Brassica napus* L.)^20^.

When evaluating the role of important amino acids for *Aphis fabae* Scopoli growth, alanine and proline were considered primarily phagostimulants, and serine also stimulated the (artificial) diet intake^44^. When histidine, methionine, threonine, valine, and possibly tryptophan, were lacking in artificial diets to test different clones of *A. fabae* nutritional requirements, its individual fitness was impaired^19^. The lack of histidine, isoleucine or methionine reduced the feeding rate of *M. persicae*, decreasing its growth rate^45^. Aphid feeding behaviour, development, fecundity and size could be impaired on N-deficient plants^46–50^. Therefore, the reduction in the content of some amino acids could have altered *A. gossypii* feeding requirements and partially explain the negative effect on aphid weight and colony growth under eCO_2_.

Accordingly to our results, *Rhopalosiphum maidis* Fitch decreased its body weight, fecundity and intrinsic population growth rate when reared on barley under eCO_2_ due to a significant reduction in crude protein, total amino acids and most of the free amino acids concentrations^24^. However, our results differ from those of Jiang et al*.*^28^, in which *A. gossypii* increased its fecundity, body weight and population abundance under eCO_2_, due to an increase in free amino acids and soluble proteins in cotton plants. To explain the divergent aphid performance, we should take into account: (1) the different host plant (melon vs cotton), (2) different clones of the same aphid species may have diverse amino acids requirements^19^, (3) the variation in the pattern of essential amino acids synthetized by the bacterial endosymbiont *Buchnera* between the different aphid clones^19,27^.

Unlike general predictions, a tendency of increase the relative abundance of essential amino acids for aphids was observed in barley phloem under eCO_2_, subsequently improving the performance of *R. padi*^22^. This cereal aphid also increased its relative growth rate in spring wheat under eCO_2_ due to an increase in most of all the individual amino acids concentrations in phloem sap^20^. Furthermore, eCO_2_ could differently change the content in foliar amino acids depending on the crop resistance to aphids, reducing the content in moderate resistant genotypes and increasing the content in resistant genotypes^23^.

Plant non-structural carbohydrates, such as starch and soluble sugars, usually increased under eCO_2_^7^. Among soluble sugars, sucrose is not only a key phagostimulant for herbivorous insects, but is also important for aphid growth and development. Thus, the increase in sucrose levels in host plants could potentially enhance aphid performance^51^. However, under eCO_2_ the rising in carbohydrates content can dilute the N nutrients required and finally counteract the positive effects of their increase^7^. This unbalance between carbohydrates and amino acids could increase aphids consumption rates due to compensatory feeding, finally increasing the plant damage^7,11^.

The eCO_2_-effect on the content of each soluble sugar depends on the host plant and could modify aphid performance differently. Elevated CO_2_ increase the concentration of fructose, mannitol and trehalose in wheat^11^ and this change in host plant quality could have produced the increase in *R. padi* weight^27^. In barley, the total soluble sugar and glucose, fructose and sucrose contents were not affected by eCO_2_, but a reduction in crude protein and amino acids content, could have influenced aphid feeding, leading to a decrease in *R. maidis* fresh body weight, fecundity and intrinsic population growth rate^24^. Due to CO_2_ enrichment, fructose and glucose concentrations increased in spring wheat but sucrose remains unchanged compared to aCO_2_, and together with a significant increase in individual amino acids concentrations, positively affected *R. padi* relative growth rate. Sucrose and individual amino acids did not change significantly their concentrations in oilseed rape under eCO_2_, finally negatively affected *M. persicae* relative growth rate^20^.

In our study, eCO_2_ significantly increased sucrose content on melon leaves and stems. Galactose, maltose and trehalose, aphid feeding stimulants as sucrose^52^ also increased their content in melon stems under eCO_2_. However, the concentrations of sugar alcohols mannitol, sorbitol and xylitol were reduced in melon plants under eCO_2_. Mannitol and sorbitol are organic osmolytes that protect aphids and whiteflies from osmotic stress and not-optimal developmental temperatures^53^. Therefore, a reduction in these polyols and some essential amino acids in melon leaves under eCO_2_, could potentially have impaired *A. gossypii* performance, decreasing aphid weight and the colony growth rate.

Our research showed how changes in plant biochemistry due to eCO_2_ have negatively affected *A. gossypii* performance. However, it is difficult to generalize the effect of eCO_2_ for phloem-feeders^12^, because their responses to eCO_2_ are heterogeneous and, in the case of aphids, the effects could be species-specific^17,54^ or even genotype-specific^23,43^. In fact, aphid populations under eCO_2_ could decrease, in accordance with our results^13,17,30,31,42,55,56^, but also increase^17,55^, or even being unaffected by eCO_2_^17^. In contrast, other insect feeding guilds responds more homogenously to eCO_2_. For instance, due to the dilution of N under eCO_2_, chewing insects show compensatory feeding, increasing their food consumption due to the lower food quality^14^. While leaf-chewers do not seem to have adverse effects on development and pupal weight under eCO_2_^14^, leaf-miners decrease their abundance and increase their development time^7^.

We observed that the acclimation period did not significantly affect melon C and N concentration and biomass. However, the differences between eCO_2_ and aCO_2_ observed under 6 weeks of acclimation were usually higher than under 2 weeks. The content of some amino acids and carbohydrates significantly increased after 6 weeks of acclimation compared to 2 weeks. Furthermore, the difference in F1 and F2 aphid biomass between eCO_2_ and aCO_2_ was greater under 6 weeks of acclimation than under 2 weeks. In general, the effects of CO_2_ on plants and aphids were higher under longer exposure; although a short period of previous plant acclimation to CO_2_ could be enough to detect eCO_2_ effects on aphid biomass. Klaiber et al.^30,31^ also showed that changes in plant and aphids were higher after longer exposure of plants to eCO_2_.

In our study, we observed differences in some amino acids and carbohydrates depending on the plant part analysed (stems or leaves) under eCO_2_. This could indicate differences in the aphid niche establishment and the consequent plant colonization^36^. Further research analysing sequential sampling at distinct melon growth stages or after different moments of plant colonization by aphids could provide in-depth information about how aphid infestation could lead to change in plant biochemistry under eCO_2_ and the subsequent impacts on plant-aphid interactions^36,57^.

In conclusion, although the change in nutritional quality of melon plants under eCO_2_ has damaged *A. gossypii* performance, and this could be thinkable as positive for pest control, the changes in foliar amino acids and carbohydrates content could make plants more palatable for other herbivore insects, or even produce a different effect on other aphid species, even on other aphid clones, which also feed on cucurbits. Therefore, further research is needed to elucidate the effect of eCO_2_ on melon crop and its associated herbivorous insects, ideally analysed in open-chambers or in a free air CO_2_ enrichment facility in order to generate more realistic predictions about how climate change affects trophic interactions in agroecosystems.

## Supplementary Information

Supplementary Information

## Data Availability

The datasets generated during the current study are available from the corresponding author on reasonable request.
