# Supplementary material for: Changes in melon plant phytochemistry impair Aphis gossypii growth and weight under elevated CO2
Source: Sci Rep. 2021 Jan 26;11:2186. doi: 10.1038/s41598-021-81167-x (PMC7838277; doi:10.1038/s41598-021-81167-x)
Supplement: Supplementary file 1 — Supplementary Information [file 41598_2021_81167_MOESM1_ESM.docx]

**Scientific Reports**

**Supplementary information**

**Changes in melon plant phytochemistry impair *Aphis gossypii* growth and weight under elevated CO_2_**

**Ana Moreno-Delafuente^1^, Ignacio Morales^1^, Elisa Garzo^2^, Alberto Fereres^2,3^, Elisa Viñuela^1,3^, Pilar Medina^1,3,*^**

^1^Unidad de Protección de Cultivos, Departamento de Producción Agraria, Escuela Técnica Superior de Ingeniería Agronómica, Alimentaria y de Biosistemas, Universidad Politécnica de Madrid, Madrid, 28040, Spain

^2^Insectos Vectores de Patógenos de Plantas, Departamento de Protección Vegetal, Instituto de Ciencias Agrarias, Consejo Superior de Investigaciones Científicas, Madrid, 28006, Spain

^3^Associate Unit IVAS (CSIC-UPM): Control of Insect Vectors of Viruses in Horticultural Sustainable Systems, Madrid, Spain

* pilar.medina@upm.es

**Supplementary Table S1. Melon carbon and nitrogen concentration means and summary of statistical analysis.** Two-way ANOVA for the effect of CO_2_ concentration, acclimation period and their interaction on Carbon (C) concentration, Nitrogen (N) concentration, C:N ratio and biomass (dry weight) in leaves and stems of melon plants (C, N, C:N, n = 9; biomass, n = 10)

|  | **Parameter** | **A.P. ^a^** | **CO_2_ ^b^** | | **Mean** | **SED^c^** | **P-value** | | |
| --- | --- | --- | --- | --- | --- | --- | --- | --- | --- |
|  |  |  | **elevated** | **ambient** |  |  | **A.P.** | **CO_2_** | **A.P. x CO_2_** |
| **Leaf** | **C (%)** | **2 w** | 39.199 | 39.858 | 39.528 | 1.515 | 0.618 | 0.959 | 0.630 |
|  |  | **6 w** | 39.172 | 38.357 | 38.765 |  |  |  |  |
|  |  | **Mean** | 39.185 | 39.108 |  |  |  |  |  |
|  | **N (%)** | **2 w** | 5.835 | 6.575 | 6.205 | 0.232 | 0.206 | **0.001** | 0.675 |
|  |  | **6 w** | 5.437 | 6.374 | 5.905 |  |  |  |  |
|  |  | **Mean** | 5.636 b | 6.474 a |  |  |  |  |  |
|  | **C:N** | **2 w** | 6.799 | 6.104 | 6.451 | 0.349 | 0.532 | **0.008** | 0.435 |
|  |  | **6 w** | 7.323 | 6.069 | 6.696 |  |  |  |  |
|  |  | **Mean** | 7.061 a | 6.086 b |  |  |  |  |  |
|  | **Biomass (g)** | **2 w** | 6.070 | 4.594 | 5.332 | 0.374 | 0.107 | **<0.001** | 0.680 |
|  |  | **6 w** | 5.607 | 3.820 | 4.713 |  |  |  |  |
|  |  | **Mean** | 5.839 a | 4.207 b |  |  |  |  |  |
| **Stem** | **C (%)** | **2 w** | 37.784 | 38.430 | 38.107 | 1.211 | 0.693 | 0.659 | 0.335 |
|  |  | **6 w** | 38.487 | 36.762 | 37.624 |  |  |  |  |
|  |  | **Mean** | 38.135 | 37.596 |  |  |  |  |  |
|  | **N (%)** | **2 w** | 4.993 | 5.356 | 5.175 | 0.215 | 0.135 | 0.069 | 0.850 |
|  |  | **6 w** | 4.623 | 5.068 | 4.846 |  |  |  |  |
|  |  | **Mean** | 4.808 | 5.212 |  |  |  |  |  |
|  | **C:N** | **2 w** | 7.690 | 7.291 | 7.491 | 0.421 | 0.364 | 0.065 | 0.343 |
|  |  | **6 w** | 8.483 | 7.273 | 7.878 |  |  |  |  |
|  |  | **Mean** | 8.087 | 7.282 |  |  |  |  |  |
|  | **Biomass (g)** | **2 w** | 3.404 | 2.244 | 2.824 | 0.260 | 0.164 | **<0.001** | 0.859 |
|  |  | **6 w** | 3.727 | 2.660 | 3.193 |  |  |  |  |
|  |  | **Mean** | 3.566 a | 2.452 b |  |  |  |  |  |
| ^a^ Acclimation period (A.P.): 2 weeks (2w) or 6 weeks (6w)  ^b^ CO_2_ concentration: elevated CO_2_ (700 ppm) or ambient CO_2_ (400 ppm) | | | | | | | | | |
| ^c^ SED: Standard error of the difference between the factors’ means | | | | | | | | | |
| Different lower case letters within rows indicate differences between CO_2_ concentrations, while no differences between acclimation periods were detected. (P ≤ 0.05) by statistical tests: Two-way ANOVA and LSD. Melon leaf C:N ratio data were transformed by log (x+1). | | | | | | | | | |

Supplementary Table S2. Melon amino acids content means and summary of statistical analysis. Two-way ANOVA for the effect of CO_2_ concentration, acclimation period and their interaction on individual amino acids content (nmol/g dry weight) in leaves and stems of melon plants (n = 6, whenever possible)

|  |  | **Melon leaf** | | | | | | |  | **Melon stem** | | | | | | |
| --- | --- | --- | --- | --- | --- | --- | --- | --- | --- | --- | --- | --- | --- | --- | --- | --- |
| **Amino acid** | **A.P.^a^** | **CO_2_^b^** | | | **SED^c^** | **P-value** | | |  | **CO_2_** | | | **SED^c^** | **P-value** | | |
|  |  | **elevated** | **ambient** | **Mean** |  | **A.P.** | **CO_2_** | **A.P. x CO_2_** |  | **elevated** | **ambient** | **Mean** |  | **A.P.** | **CO_2_** | **A.P. x CO_2_** |
| **Alanine** | **2 w** | 55.29 | 91.72 | 73.51 | 19.96 | 0.933 | **0.031** | 0.615 |  | 32.95 | 40.93 | 36.94 | 17.73 | 0.637 | 0.658 | 0.325 |
|  | **6 w** | 43.38 | 100.26 | 71.82 |  |  |  |  |  | 54.00 | 28.82 | 41.41 |  |  |  |  |
|  | **Mean** | 49.33 b | 95.99 a |  |  |  |  |  |  | 43.47 | 34.87 |  |  |  |  |  |
| **Asparagine** | **2 w** | 10.76 | 31.39 | 21.07 | 6.79 | 0.252 | **0.010** | 0.825 |  | 11.04 b | 33.04 Aa | 22.04 | 5.69 | 0.237 | 0.282 | **0.015** |
|  | **6 w** | 9.72 | 27.26 | 18.49 |  |  |  |  |  | 19.70 | 10.38 B | 15.04 |  |  |  |  |
|  | **Mean** | 10.24 b | 29.32 a |  |  |  |  |  |  | 15.37 | 21.71 |  |  |  |  |  |
| **Aspartic acid** | **2 w** | 3.23 | 4.06 | 3.64 | 1.81 | 0.981 | 0.129 | 0.397 |  | 2.42 | 4.95 | 3.68 | 1.56 | 0.474 | 0.713 | 0.066 |
|  | **6 w** | 2.75 | 7.19 | 4.97 |  |  |  |  |  | 4.39 | 0.68 | 2.53 |  |  |  |  |
|  | **Mean** | 2.99 | 5.63 |  |  |  |  |  |  | 3.40 | 2.82 |  |  |  |  |  |
| **Cysteine^d^** | **2 w** | 319.13 | 424.58 | 371.86 | 145.76 | 0.509 | 0.910 | 0.556 |  |  |  |  |  |  |  |  |
|  | **6 w** | 308.32 | 236.62 | 272.47 |  |  |  |  |  | - | - | - | - | - | - | - |
|  | **Mean** | 313.72 | 330.60 |  |  |  |  |  |  |  |  |  |  |  |  |  |
| **Glutamate^d^** | **2 w** | 41.12 | 79.27 | 60.19 | 31.33 | 0.789 | 0.074 | 0.645 |  |  |  |  |  |  |  |  |
|  | **6 w** | 43.40 | 133.75 | 88.57 |  |  |  |  |  | - | - | - | - | - | - | - |
|  | **Mean** | 42.26 | 106.51 |  |  |  |  |  |  |  |  |  |  |  |  |  |
| **Glutamine** | **2 w** | 56.61 | 117.59 | 87.10 | 42.18 | 0.335 | 0.177 | 0.733 |  | 388.58 | 300.13 | 344.36 | 101.54 | 0.237 | 0.207 | 0.660 |
|  | **6 w** | 135.15 | 179.41 | 157.28 |  |  |  |  |  | 309.06 | 129.39 | 219.22 |  |  |  |  |
|  | **Mean** | 95.88 | 148.50 |  |  |  |  |  |  | 348.82 | 214.76 |  |  |  |  |  |
| **Glycine** | **2 w** | 47.54 | 111.73 | 79.63 | 28.70 | 0.289 | **0.002** | 0.396 |  | 21.53 | 48.29 | 34.91 | 11.36 | 0.698 | 0.300 | 0.054 |
|  | **6 w** | 25.73 | 137.67 | 81.70 |  |  |  |  |  | 32.32 | 20.69 | 26.50 |  |  |  |  |
|  | **Mean** | 36.63 b | 124.70 a |  |  |  |  |  |  | 26.93 | 34.49 |  |  |  |  |  |
| **Histidine** | **2 w** | 5.52 | 5.26 | 5.39 | 1.51 | 0.287 | 0.909 | 0.779 |  | 7.19 | 5.98 | 6.58 | 2.17 | 0.936 | 0.724 | 0.596 |
|  | **6 w** | 6.74 | 7.35 | 7.04 |  |  |  |  |  | 6.52 | 6.60 | 6.56 |  |  |  |  |
|  | **Mean** | 6.13 | 6.30 |  |  |  |  |  |  | 6.86 | 6.29 |  |  |  |  |  |
| **Isoleucine** | **2 w** | 0.07 | 0.18 | 0.12 | 0.03 | 0.485 | **0.018** | 0.367 |  | 0.14 | 0.18 | 0.16 | 0.08 | 0.452 | 0.522 | 0.822 |
|  | **6 w** | 0.12 | 0.18 | 0.15 |  |  |  |  |  | 0.16 | 0.27 | 0.22 |  |  |  |  |
|  | **Mean** | 0.10 b | 0.18 a |  |  |  |  |  |  | 0.15 | 0.23 |  |  |  |  |  |
| **Lysine** | **2 w** | 3.74 | 6.86 | 5.30 | 4.09 | 0.171 | **0.017** | 0.146 |  | 6.38 | 10.16 | 8.27 | 2.32 | 0.712 | 0.632 | 0.051 |
|  | **6 w** | 5.40 | 31.74 | 18.57 |  |  |  |  |  | 10.42 | 4.37 | 7.40 |  |  |  |  |
|  | **Mean** | 4.57 b | 19.30 a |  |  |  |  |  |  | 8.40 | 7.27 |  |  |  |  |  |
|  |  |  |  |  |  |  |  |  |  |  |  |  |  |  |  |  |
| **Methionine^d^** | **2 w** | 2.52 | 2.52 | 2.52 B | 0.91 | **0.039** | 0.699 | 0.642 |  |  |  |  |  |  |  |  |
|  | **6 w** | 4.20 | 5.63 | 4.91 A |  |  |  |  |  | - | - | - | - | - | - | - |
|  | **Mean** | 3.36 | 4.07 |  |  |  |  |  |  |  |  |  |  |  |  |  |
| **Phenylalanine** | **2 w** | 18.68 | 20.80 | 19.74 | 9.04 | 0.362 | 0.086 | 0.155 |  | 60.74 | 38.91 | 49.83 | 21.88 | 0.632 | 0.151 | 0.591 |
|  | **6 w** | 19.43 | 52.46 | 35.95 |  |  |  |  |  | 12.39 | 63.97 | 38.18 |  |  |  |  |
|  | **Mean** | 19.06 | 36.63 |  |  |  |  |  |  | 36.57 | 51.44 |  |  |  |  |  |
| **Proline** | **2 w** | 2.13 | 3.68 | 2.91 | 0.84 | 0.559 | 0.052 | 0.919 |  | 1.68 | 2.73 | 2.20 | 0.47 | 0.148 | 0.212 | 0.307 |
|  | **6 w** | 2.49 | 3.87 | 3.18 |  |  |  |  |  | 1.39 | 1.65 | 1.52 |  |  |  |  |
|  | **Mean** | 2.31 | 3.78 |  |  |  |  |  |  | 1.53 | 2.19 |  |  |  |  |  |
| **Serine** | **2 w** | 3.80 | 6.50 | 5.15 | 1.12 | 0.234 | **0.000** | 0.087 |  | 2.28 | 2.58 | 2.43 | 0.53 | 0.777 | 0.459 | 0.261 |
|  | **6 w** | 2.28 | 8.21 | 5.24 |  |  |  |  |  | 2.81 | 1.79 | 2.30 |  |  |  |  |
|  | **Mean** | 3.04 b | 7.35 a |  |  |  |  |  |  | 2.55 | 2.18 |  |  |  |  |  |
| **Threonine** | **2 w** | 269.59 | 444.70 | 357.14 | 75.68 | 0.332 | **0.011** | 0.627 |  | 137.17 | 171.29 | 154.23 A | 43.81 | **0.029** | 0.451 | 0.992 |
|  | **6 w** | 157.02 | 406.73 | 281.88 |  |  |  |  |  | 33.82 | 67.09 | 50.46 B |  |  |  |  |
|  | **Mean** | 213.31 b | 425.72 a |  |  |  |  |  |  | 85.50 | 119.19 |  |  |  |  |  |
| **Tryptophan** | **2 w** | 9.55 | 11.88 | 10.71 | 2.30 | 0.776 | 0.625 | 0.613 |  | 6.43 | 11.92 | 9.17 | 2.46 | 0.321 | **0.039** | 0.989 |
|  | **6 w** | 11.40 | 11.36 | 11.38 |  |  |  |  |  | 3.96 | 9.38 | 6.67 |  |  |  |  |
|  | **Mean** | 10.47 | 11.62 |  |  |  |  |  |  | 5.19 b | 10.65 a |  |  |  |  |  |
| **Tyrosine** | **2 w** | 233.67 | 248.73 | 241.20 | 38.62 | 0.526 | 0.831 | 0.934 |  | 632.58 | 213.59 | 423.08 | 141.93 | 0.361 | **0.032** | 0.555 |
|  | **6 w** | 229.15 | 243.77 | 236.46 |  |  |  |  |  | 674.91 | 462.88 | 568.90 |  |  |  |  |
|  | **Mean** | 231.41 | 246.25 |  |  |  |  |  |  | 653.75 a | 338.23 b |  |  |  |  |  |
| **Valine** | **2 w** | 1.54 | 2.46 | 2.00 | 0.67 | 0.972 | **0.032** | 0.445 |  | 0.83 | 1.81 | 1.32 | 0.44 | 0.806 | 0.176 | 0.233 |
|  | **6 w** | 1.08 | 3.37 | 2.22 |  |  |  |  |  | 0.98 | 1.19 | 1.08 |  |  |  |  |
|  | **Mean** | 1.31 b | 2.92 a |  |  |  |  |  |  | 0.91 | 1.50 |  |  |  |  |  |
| ^a^ Acclimation period (A.P.): 2 weeks (2w) or 6 weeks (6w) | | | | | | | | | | | | | | | | |
| ^b^ CO_2_ concentration: elevated CO_2_ (700 ppm) or ambient CO_2_ (400 ppm) | | | | | | |  |  |  |  |  |  |  |  |  |  |
| ^c^ SED: Standard error of the difference between the factors’ means | | | | | | | | |  |  |  |  |  |  |  |  |
| ^d^ Compounds only detected in less than 3 repetitions per treatment on melon stem samples, not possible to perform statistical analysis. | | | | | | | | | | | | | |  |  |  |
| Different lower case letters within rows indicate differences between CO_2_ concentrations, while different upper case letters within columns indicate differences between acclimation periods (P ≤ 0.05) by statistical tests: Two-way ANOVA and LSD for pairwise comparison. Some variables were transformed by log (x+1) or sqrt (x+0.5) to achieve normality and homoscedasticity. | | | | | | | | | | | | | | | | |

**Supplementary Table S3.** **Melon carbohydrates content means and summary of statistical analysis.** Two-way ANOVA for the effect of CO_2_ concentration, acclimation period and their interaction on carbohydrates content (nmol/g dry weight) in leaves and stems of melon plants (n = 6, whenever possible)

|  | |  | **Melon leaf** | | | | | | |  | **Melon stem** | | | | | | | | |  |
| --- | --- | --- | --- | --- | --- | --- | --- | --- | --- | --- | --- | --- | --- | --- | --- | --- | --- | --- | --- | --- |
| **Carbohydrate** | | **A.P.^a^** | **CO_2_^b^** | | | **SED^c^** | **P-value** | | |  | **CO_2_** | | |  | | **P-value** | | | |  |
|  |  |  | **elevated** | **ambient** | **Mean** |  | **A.P.** | **CO_2_** | **A.P. x CO_2_** |  | **elevated** | **ambient** | **Mean** | | **SED^c^** | | **A.P.** | **CO_2_** | **A.P. x CO_2_** | |
| **Mono-saccharides** | **Fructose^d^** | **2 w** | 0.50 | 0.76 | 0.63 B | 0.37 | **0.034** | 0.701 | 0.294 |  |  |  |  | |  | |  |  |  | |
|  |  | **6 w** | 1.79 | 1.24 | 1.52 A |  |  |  |  |  | - | - | - | | - | | - | - | - | |
|  |  | **Mean** | 1.15 | 1.00 |  |  |  |  |  |  |  |  |  | |  | |  |  |  | |
|  | **Galactose** | **2 w** | 14.60 | 18.90 | 16.75 | 5.87 | 0.366 | 0.424 | 0.931 |  | 76.949 | 19.549 | 48.25 B | | 27.331 | | **0.004** | **0.009** | 0.301 | |
|  |  | **6 w** | 19.54 | 24.87 | 22.21 |  |  |  |  |  | 151.272 | 74.733 | 113.00 A | |  | |  |  |  | |
|  |  | **Mean** | 17.07 | 21.89 |  |  |  |  |  |  | 114.11 a | 47.14 b |  | |  | |  |  |  | |
|  | **Glucose** | **2 w** | 24.73 | 26.74 | 25.73 | 2.61 | 0.441 | 0.638 | 0.772 |  | 36.187 | 29.943 | 33.065 | | 3.582 | | 0.197 | 0.320 | 0.481 | |
|  |  | **6 w** | 27.55 | 28.03 | 27.79 |  |  |  |  |  | 28.811 | 27.726 | 28.269 | |  | |  |  |  | |
|  |  | **Mean** | 26.14 | 27.39 |  |  |  |  |  |  | 32.499 | 28.834 |  | |  | |  |  |  | |
| **Di-saccharides** | **Maltose** | **2 w** | 5.72 | 0.93 | 3.33 B | 12.44 | **0.047** | 0.881 | 0.572 |  | 11.214 | 0.750 | 5.982 | | 3.490 | | 0.141 | **0.004** | 0.394 | |
|  |  | **6 w** | 35.25 | 14.72 | 24.98 A |  |  |  |  |  | 7.138 | 0.719 | 3.928 | |  | |  |  |  | |
|  |  | **Mean** | 20.49 | 7.82 |  |  |  |  |  |  | 9.18 a | 0.73 b |  | |  | |  |  |  | |
|  | **Sucrose** | **2 w** | 1.64 | 0.69 | 1.17 | 0.32 | 0.121 | **0.017** | 0.775 |  | 3.183 | 0.957 | 2.070 | | 1.224 | | 0.864 | **<0.001** | 0.942 | |
|  |  | **6 w** | 2.08 | 1.31 | 1.69 |  |  |  |  |  | 4.750 | 0.708 | 2.729 | |  | |  |  |  | |
|  |  | **Mean** | 1.86 a | 1.00 b |  |  |  |  |  |  | 3.97 a | 0.83 b |  | |  | |  |  |  | |
|  | **Trehalose** | **2 w** | 5.69 | 1.28 | 3.49 B | 19.42 | **0.048** | 0.807 | 0.799 |  | 12.888 | 0.718 | 6.803 | | 4.029 | | 0.147 | **0.005** | 0.364 | |
|  |  | **6 w** | 51.82 | 14.74 | 33.28 A |  |  |  |  |  | 7.766 | 0.722 | 4.244 | |  | |  |  |  | |
|  |  | **Mean** | 28.75 | 8.01 |  |  |  |  |  |  | 10.33 a | 0.72 b |  | |  | |  |  |  | |
| **Polyols (sugar alcohols)** | **Mannitol^d^** | **2 w** | 42.79 | 59.97 | 51.38 | 6.93 | 0.059 | **0.001** | 0.158 |  |  |  |  | |  | |  |  |  | |
|  |  | **6 w** | 18.33 | 56.10 | 37.21 |  |  |  |  |  | - | - | - | | - | | - | - | - | |
|  |  | **Mean** | 30.56 b | 58.04 a |  |  |  |  |  |  |  |  |  | |  | |  |  |  | |
|  | **Sorbitol** | **2 w** | 16.48 | 21.15 | 18.82 | 3.57 | 0.950 | **0.031** | 0.314 |  | 31.782 | 16.299 | 24.041 | | 6.315 | | 0.899 | 0.231 | 0.537 | |
|  |  | **6 w** | 12.55 | 24.63 | 18.59 |  |  |  |  |  | 27.023 | 24.303 | 25.663 | |  | |  |  |  | |
|  |  | **Mean** | 14.52 b | 22.89 a |  |  |  |  |  |  | 29.403 | 20.301 |  | |  | |  |  |  | |
|  | **Xylitol** | **2 w** | 5.62 | 23.08 | 14.35 | 7.73 | 0.629 | **0.026** | 0.507 |  | 8.049 | 15.158 | 11.604 | | 6.313 | | 0.508 | 0.268 | 0.553 | |
|  |  | **6 w** | 13.29 | 27.47 | 20.38 |  |  |  |  |  | 8.091 | 4.805 | 6.448 | |  | |  |  |  | |
|  |  | **Mean** | 9.46 b | 25.28 a |  |  |  |  |  |  | 8.070 | 9.982 |  | |  | |  |  |  | |
|  |  |  |  |  |  |  |  |  |  |  |  |  |  | |  | |  |  |  | |
| **Gluconic acid** | **Gluconate** | **2 w** | 29.98 | 26.33 | 28.16 | 2.86 | 0.868 | 0.352 | 0.753 |  | 44.305 | 24.544 | 34.424 | | 7.471 | | 0.937 | 0.210 | 0.469 | |
|  |  | **6 w** | 29.55 | 27.73 | 28.64 |  |  |  |  |  | 37.886 | 32.049 | 34.968 | |  | |  |  |  | |
|  |  | **Mean** | 29.77 | 27.03 |  |  |  |  |  |  | 41.095 | 28.297 |  | |  | |  |  |  | |
| ^a^ Acclimation period (A.P.): 2 weeks (2w) or 6 weeks (6w) | | | | | | | | | | | | | | | | | | | | |
| ^b^ CO_2_ concentration: elevated CO_2_ (700 ppm) or ambient CO_2_ (400 ppm) | | | | | | | | | | | | | | | | | | | | |
| ^c^ SED: Standard error of the difference between the factors’ means | | | | | | | | | | | | | | | | | | | | |
| ^d^ Compounds only detected in less than 3 repetitions per treatment on melon stem samples, not possible to perform statistical analysis. | | | | | | | | | | | | | | | | | | | | |
| Different lower case letters within rows indicate differences between CO_2_ concentrations, while different upper case letters within columns indicate differences between acclimation periods (P ≤ 0.05) by statistical tests: Two-way ANOVA and LSD for pairwise comparison. Some variables were transformed by log (x+1) or sqrt (x+0.5) to achieve normality and homoscedasticity. | | | | | | | | | | | | | | | | | | | | |

Supplementary Table S4. Summary of Student *t*-test results for *Aphis gossypii* abundance and growth rate. Number of adults, nymphs and colony growth rates (mean ± SE) of *Aphis gossypii* developed under ambient (400 ppm) or elevated (700 ppm) CO_2_, on melon plants acclimated for two weeks to the respective CO_2_ concentration. Growth rates were calculated weekly (day 14 as the difference in the number of aphids on day 14 compared to day 7; and day 21, comparing the number of aphids on day 21 to day 14). Ten (n) *A. gossypii* colonies per CO_2_ concentration. P-values based on Student *t*-test (P ≤ 0.05)

| Growth rate | Period | ambient CO_2_ | | | elevated CO_2_ | | | t | df | P-value |
| --- | --- | --- | --- | --- | --- | --- | --- | --- | --- | --- |
| Adults | 7d-  14d | 43.80 | ± | 4.97 | 36.00 | ± | 3.07 | -1.334 | 18 | 0.199 |
| Nymphs |  | 253.40 | ± | 21.66 | 214.10 | ± | 16.72 | -1.436 | 18 | 0.168 |
| Colony |  | 297.20 | ± | 24.55 | 250.10 | ± | 17.92 | -1.550 | 18 | 0.139 |
| Adults | 14d-21d | 443.70 | ± | 66.34 | 425.50 | ± | 41.21 | -0.233 | 18 | 0.818 |
| Nymphs |  | 7866.90 | ± | 535.19 | 5969.90 | ± | 465.09 | -2.675 | 18 | **0.015** |
| Colony |  | 8310.60 | ± | 587.87 | 6395.40 | ± | 498.15 | -2.486 | 18 | **0.023** |
